# Mortality Profile of Deaths Related to Infective Endocarditis in Brazil and Regions: A Population-Based Analysis of Death Records

**DOI:** 10.3390/tropicalmed9120291

**Published:** 2024-11-29

**Authors:** João Vitor Fazzio de Andrade Cordeiro, Letícia Martins Raposo, Paulo Henrique Godoy

**Affiliations:** 1 School of Medicine and Surgery, Federal University of the State of Rio de Janeiro, Rua Silva Ramos, 32, Tijuca, Rio de Janeiro 20270-330, Brazil; fazziojv@edu.unirio.br; 2 Department of Quantitative Methods, School of Mathematics, Federal University of the State of Rio de Janeiro, Av. Pasteur, 296—Urca, Rio de Janeiro 22290-240, Brazil; leticia.raposo@uniriotec.br; 3 Department of Specialized Medicine—Cardiology Discipline, School of Medicine and Surgery, Federal University of the State of Rio de Janeiro, Rua Silva Ramos, 32, Tijuca, Rio de Janeiro 20270-330, Brazil

**Keywords:** endocarditis, epidemiological profile, mortality, upper middle-income countries, Brazil

## Abstract

Background: Studies of infective endocarditis (IE) are generally limited to institutions, underlining the need for more comprehensive epidemiological research. Objective: The aim of this study was to determine the mortality profile of IE-related deaths and related causes in Brazil at the national level and across regions. Method: We conducted a population-based study using data from the country’s mortality information system for the period 2000 to 2019. We identified IE-related deaths and related causes based on the ICD-10 codes. Cluster analysis was performed to determine the relationship between the variables. Results: There were 52,055 IE-related deaths during the study period. Deaths occurred predominantly among men and people aged between 60 and 79 years. The Southeast accounted for the largest proportion of deaths. The most frequent ICD-10 chapter mentioned in relation to IE-related deaths was diseases of the circulatory system. We identified three distinctive profiles: 1—an age of 80 years and over and women, where the most frequent chapters were endocrine, circulatory and metabolic diseases and the South and Southeast accounted for the largest proportion of deaths; 2—an age between 30 and 79 years and men, where the most frequent chapters were infectious and genitourinary diseases and the South and Southeast accounted for the largest proportion of deaths; and 3—an age between 0 and 29 years without any difference between sexes, where the most frequent chapter was diseases of the respiratory system and the North, Northeast and Midwest accounted for the largest proportion of deaths. Conclusions: The findings of the cluster analysis revealed distinctive IE-related mortality profiles, indicating regional differences.

## 1. Introduction

Infective endocarditis (IE) is defined as an infection of the endocardium, an intracardiac device or valves [1].

The epidemiological profile of the disease varies according to country. In high-income countries, it is associated with degenerative valve disease and is more frequent in middle-aged individuals [2,3]. In lower middle-income countries, low-income countries and even some upper middle-income countries such as Brazil, the leading predisposing condition is rheumatic fever with heart damage, which is prevalent in individuals in their 30s [2,3]. Brazil and Argentina have shown a reduction in the number of cases of IE associated with rheumatic fever. This may be partially explained by the use of prophylaxis in patients at risk of IE undergoing dental procedures, as recommended by the guidelines [4,5,6].

Incidence of IE varies considerably according to region and country. In recent decades, incidence rates ranging from 2 to 7.55 cases per 100,000 people have been reported in Denmark and Spain [7,8], while in North Africa and the Middle East, rates have ranged from 7.8 to 11.7 cases per 100,000 people [9]. In 2019, seven European Society of Cardiology-affiliated centers in Brazil reported 129 confirmed cases of IE. The center case rate was similar to that reported in southern European countries [10].

Despite its low prevalence, IE is a life-threatening disease associated with serious complications, such as heart failure, brain abscesses and mycotic aneurysms [1]. Therefore, IE-related mortality remains a concern, with evidence showing that rates range from 8% to 40%, depending on the study population and the assessment method used [11,12]. Mortality is heavily influenced by the incidence of complications and late diagnosis. The most frequent complications contributing to IE-related hospital deaths are sepsis and heart failure [10]. Observational studies in Brazil reported that the main complications contributing to death in patients with IE were acute renal failure, septic shock and heart failure [13,14,15]. The same studies also suggested that diseases such as diabetes mellitus and chronic kidney disease, together with advanced age, were factors associated with poor prognosis [13,14,15].

Advancing our knowledge of the factors associated with death from a disease can help improve our understanding of the development of the condition and prognosis. Studies of IE are mostly limited to institutions, underlining the need for more comprehensive epidemiological research, particularly in low- and middle-income countries. The aim of this study was therefore to determine the frequency of IE-related deaths in Brazil and across regions between 2000 and 2019 and analyze the mortality profile, focusing on other related causes and sociodemographic variables.

## 2. Materials and Methods

We conducted an observational time series analysis of IE-related deaths using data from the country’s mortality information system (SIM), run by the national health service’s Department of Informatics (DATASUS). The data were extracted using R’s microdatasus package version 2.1.0 [16] based on the following codes listed in the International Statistical Classification of Diseases and Related Health Problems 10th Revision (ICD-10) [17]: I 33—acute and subacute endocarditis; I 33.0—acute and subacute infective endocarditis; I 33.9—acute endocarditis, unspecified; I 38—endocarditis, valve unspecified; I 39—endocarditis and heart valve disorders in diseases classified elsewhere; I 39.0—mitral valve disorders in diseases classified elsewhere; I 39.1—aortic valve disorders in diseases classified elsewhere; I 39.2—tricuspid valve disorders in diseases classified elsewhere; I 39.3—pulmonary valve disorders in diseases classified elsewhere; I 39.4—multiple valve disorders in diseases classified elsewhere; I 39.8—endocarditis, valve unspecified, in diseases classified elsewhere.

The SIM database includes a column showing the underlying cause of death, which is generated from the information contained in lines A, B, C and D (part I) of the death certificate, and other columns corresponding to lines A, B, C, D and part II of the death certificate. The latter includes other significant conditions contributing to death but not resulting in the underlying cause given in part I. To avoid underestimation of IE-related deaths, when IE was not mentioned in the underlying cause column, a search was also performed of the columns corresponding to lines A, B, C, D and part II of the death certificate. This also avoided duplication of IE-related deaths where IE was mentioned in both the underlying cause column and one of the columns corresponding to lines A, B, C, D and part II of the death certificate.

Related causes mentioned in the underlying cause column and columns corresponding to lines A, B, C, D and part II of the death certificate were also included for all IE-related deaths and categorized into the ICD-10 chapters. Chapters accounting for less than 5% of overall deaths were excluded from the analysis.

The following variables were analyzed: (i) period (2000–2004, 2005–2009, 2010–2014, 2015–2019); (ii) age group (0–9, 10–29, 30–59, 60–79, 80 and over); (iii) sex (male, female); (iv) race/color (Asian, white, indigenous, brown, black); (v) marital status (single, married/consensual union, separated, widowed); (vi) education level (no education, 1–7 years, 8–11 years, 12 or more years); (vii) place of death (home, hospital/other health facility, other); (viii) ICD-10 chapter; and (ix) region (North, Northeast, Midwest, Southeast and South). Figure S1 in the Supplementary Material presents a map of Brazil showing the five regions and relevant demographic indicators (average population, area and Human Development Index).

### Cases with Missing Data Were Excluded

Absolute frequencies and percentages were used to describe the qualitative variables. The association between the variables and regions was assessed using Pearson’s chi-squared test. The proportions of deaths represented by each ICD-10 chapter were compared across regions using 95% confidence intervals (95% CI). Since analysis can be very time-consuming, cluster analysis was performed using a representative sample of 5000 observations for each period, totaling 20,000 observations.

Multiple correspondence analysis (MCA) was performed using R’s FactoMineR package version 2.8 [18], followed by hierarchical clustering of the principal components detected by MCA using Ward’s agglomeration method with Euclidean distance. The optimum number of clusters was determined automatically and the proportions of the categories in the clusters were compared using the V test based on hypergeometric distribution [19]. The clusters were interpreted using the Cla/Mod metric (the proportion of individuals within a specific class (cluster)) and Mod/Cla metric (the proportion of individuals within a class with a specific modality (cluster)) (Table S1—Supplementary Material). To provide a visual comparison of characteristics between clusters, a heat map was created in R’s pheatmap package [20] using a color scale to represent the frequency of characteristics in each cluster. The characteristics within the heat map were ordered using hierarchical clustering to provide an intuitive representation of associations between variables. All analyses were performed using R version 4.3.2, adopting a 5% significance level.

Although this study used publicly available data, the study protocol was approved by Gaffrée and Guinle University Hospital’s research ethics committee (reference code 7.051.294, 3 September 2024).

## 3. Results

### 3.1. Profile of IE-Related Deaths

A total of 52,055 IE-related deaths were recorded in Brazil between 2000 and 2019. The Southeast, the country’s most populous region (Figure S1), accounted for the highest proportion of deaths (54.00%), followed by the Northeast and South (19.06% and 15.76%, respectively). The region that accounted for the lowest proportion of deaths was the North (4.16%) (Table 1).

The 60–79-year age group accounted for the highest proportion of deaths at the national level and in the South and Southeast, while the 30–59-year age group accounted for the highest proportion in the North, Northeast and Midwest. Men accounted for a higher proportion of deaths than women nationally and across all regions (Table 1).

The findings also show racial disparities in IE-related deaths. Nationally and in the South, Southeast and Midwest, white individuals accounted for the highest proportion of deaths, while in the North and Northeast, deaths occurred predominantly among brown people. With regard to marital status, people who were married accounted for the highest proportion of deaths nationally and across all regions (Table 1).

Regarding education level, people with between 1 and 7 years of education accounted for the highest proportion of deaths nationally and across all regions. The most frequent place of occurrence of death was a hospital or other health facility nationally and across all regions. The regions with the highest proportion of deaths at home were the Midwest, South and Northeast (Table 1).

**Figure 1:**
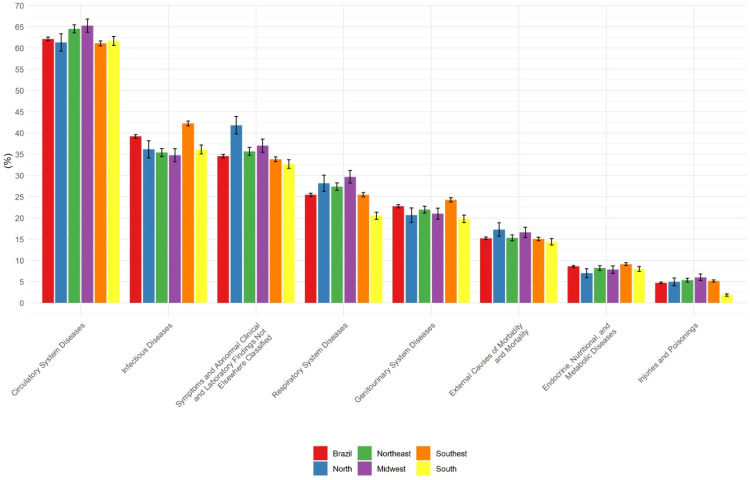
Other causes related to IE-related deaths by ICD-10 chapter. Brazil and regions, 2000 to 2019.

The most frequent causes associated with IE-related deaths at the national level were diseases of the circulatory system, with the highest rates being found in the Midwest (65.2%, 95% CI = 63.7–66.8%) and Northeast (64.5%, 95% CI = 63.5–65.4%) (Figure 1). The most frequent diseases of the circulatory system were heart failure, cerebrovascular diseases and hypertensive diseases.

The second most frequent chapter was infectious and parasitic diseases, with the highest rate being found in the Southeast (42.2%, 95% CI = 41.7–42.8%). The most frequent condition in this chapter was sepsis (unspecified organism). The second most frequent chapter was symptoms, signs and abnormal clinical and laboratory findings (not elsewhere classified), with the highest rate being found in the North (41.8%, 95% CI = 39.7–43.9%). The most frequent subcategory in this chapter was cardiogenic shock (Figure 1). Deaths from diseases of the respiratory system were also frequent but were less frequent in the South (20.5% (19.6–21.4%). The most frequent condition in this chapter was respiratory failure. The frequency of deaths from diseases of the genitourinary system at the national level was 22.7% (95% CI = 22.4–23.1%), with the rate being the highest in the Southeast (24.3%, 95% CI = 23.8–24.8%) (Figure 1). The most frequent condition in the chapter was kidney failure. The most frequent external causes of morbidity and mortality were abnormal reaction of the patient and later complication after surgery, with prosthesis implantation being the most frequent of these causes. Frequency was higher in the North (17.3%, 95% CI = 15.7–18.8%) than in the Southeast and South. The frequency of endocrine, nutritional and metabolic diseases was the highest in the Southeast. The most frequent conditions in this chapter were diabetes mellitus and malnutrition (9.1%, 95% CI = 8.8–9.4%) (Figure 1).

### 3.2. Cluster Analysis of IE-Related Deaths

The analysis identified three clusters of IE-related deaths with distinctive patient profiles.

**Figure 2:**
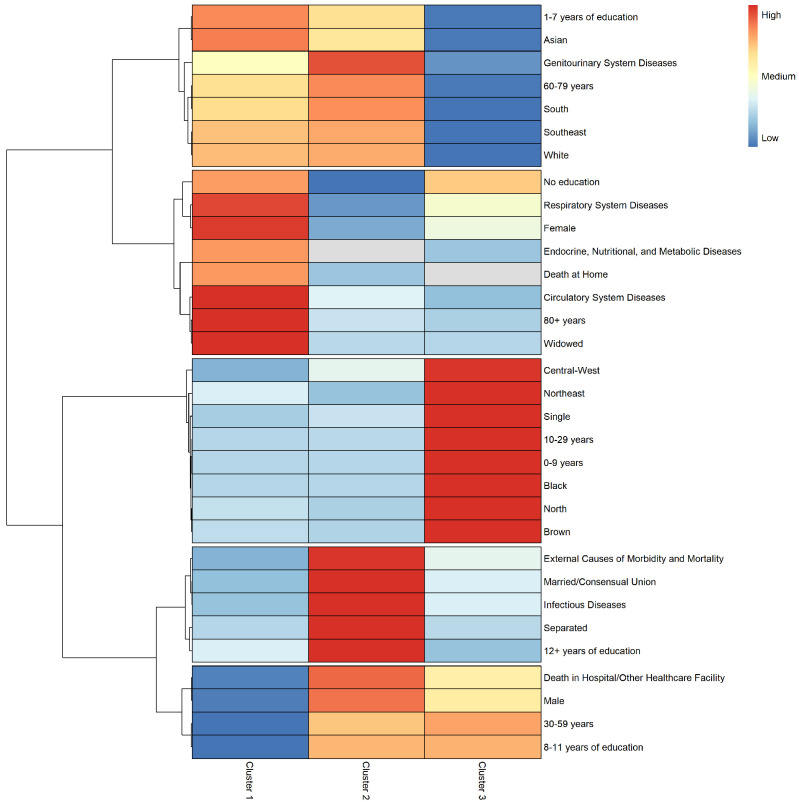
Mortality profile of IE-related deaths by cluster based on hierarchical clustering of the principal components detected by multiple correspondence analysis.

Cluster 1 was made up predominantly of individuals aged 80 years and over (52.3%) and women (79.8%), representing 72.8% and 34.7%, respectively, of all individuals in these groups in the overall sample. The percentage of white and Asian individuals and people from the Southeast and South was higher in this cluster than in the overall sample. Most of the cluster (81.8%) were widowed, corresponding to 91.9% of all widows in the overall sample. The proportion of individuals with no education and with between 1 and 7 years of education was higher in this cluster than in the overall sample. Deaths at home in this cluster (13.7% of total deaths) accounted for 41.2% of overall deaths at home. The most frequent other causes mentioned on the birth certificate were endocrine and metabolic diseases, diseases of the circulatory system and diseases of the respiratory system (Figure 2, Table S1).

Over 90% of the individuals in cluster 2 were aged between 30 and 79 years. The cluster was made up predominantly of men (68.4%), accounting for 66.7% of all men in the overall sample. The proportion of white and Asian individuals was higher in this cluster than in the overall sample. The Southeast accounted for the highest percentage of people in this cluster (62.2%), followed by the South (22.3%), representing 61.1% and 70.4%, respectively, of all individuals from these regions in the overall sample. Most individuals in this cluster were married or separated. The proportion of individuals with no education was low in this cluster. Hospital deaths accounted for 95% of all deaths in this cluster, representing 55% of all hospital deaths in the overall sample. Deaths from infectious diseases and diseases of the genitourinary system accounted for 41.8% and 26.2% of deaths in the cluster, respectively, representing 57.7% and 61.4% of overall deaths from these diseases, respectively. External causes were also frequent (Figure 2, Table S1).

Cluster 3 was characterized by a greater presence of younger age groups, with individuals aged 0–9 representing 4.5% of the deaths in the cluster and 100% of overall deaths in this age group, and the 10–29 age group accounting for 31.3% of the deaths in the cluster and 98.2% of overall deaths in this age group. Both sexes showed similar percentages and the predominant race/color in this cluster was brown and black. The percentage of individuals from the North, Northeast and Midwest was higher than in the overall sample, with the Northeast accounting for 38.9% of all deaths in the cluster. Single individuals accounted for 74.3% of deaths in the cluster, representing 74.6% of all deaths among single individuals in the overall sample. The frequency of diseases of the respiratory system was higher in this cluster than in cluster 2 and in the overall sample (Figure 2, Table S1).

## 4. Discussion

The findings reveal that there was a significant number of IE-related deaths (52,055) in Brazil during the study period. IE mortality varies considerably worldwide; however, population-based studies of the disease are scarce, especially in Latin America. The EIRA 3 study, a prospective multicenter observational study of patients with definite IE conducted in Argentina in 2018, showed that mortality remained high and similar to the rates observed in the EIRA 1 and 2 studies in 1993 and 2002, respectively [21]. A 17-year prospective population-based study conducted in Italy also showed an increasing trend in IE mortality [22]. The number of deaths found by the present study may therefore suggest an increasing trend in mortality.

The proportions of deaths across regions found by the current study are similar to those reported by Oliveira et al. in a study investigating valvular IE morbidity and mortality using DATASUS data on procedures in public hospitals during the period 2018–2021. The authors found that morbidity and mortality rates were the highest in the Southeast and lowest in the North [23]. The high proportion of IE in the Southeast may be explained by the region’s high population density and better quality of death records in this region [24,25]. This finding is similar to the results of a study investigating IE mortality during the period 2012–2021 using SIM data by David et al., who found that the Southeast accounted for the highest proportion of deaths (53.5%) [26]. Differences in data quality across states can also influence regional results. The North has the highest level of inconsistencies between the SIM and civil registration system, as demonstrated by the 2010 census [25]. Thus, the low proportion of IE-related deaths in the North may be explained by a lower disease prevalence in this region or underreporting.

The results of the current study showing that IE predominantly affects older patients (60 years and over) are consistent with those in the literature [2,3,4,5,11,12,13,14], suggesting a relationship between advanced age and fatal IE-related outcomes. The relationship between ageing and higher vulnerability to IE has been documented by previous studies showing that deaths tend to be concentrated in populations with higher age- and gender-related EI prevalence [2,3,4,5,12,13,14].

The racial disparities in deaths between regions found by the present study are consistent with differences in demographic characteristics across Brazil, with a predominance of white individuals in the Mid-South macro region and brown/black individuals in the North and Northeast [24,25]. Our findings show a higher proportion of deaths among married individuals and those with a low level of education (between 1 and 7 years) at the national level. In an ecological study of deaths from acute and subacute IE in the Northeast between 2010 and 2019 using data from the national notifiable diseases information system (SINAN), Melo et al. found a strong positive correlation between IE mortality and being male, brown, aged between 65 and 75 years, married and having completed 8 to 11 years of education [27], corroborating the influence of socio-economic factors such as education level on disease mortality.

Our findings show that the most frequent place of occurrence of death was a hospital or other health care facility. This was to be expected given that the treatment of IE is generally hospital-based so that the patient can undergo intravenous therapy. In this respect, a population-based study in Finland showed that 89.5% of IE-related deaths occurred in a hospital or other health care facility and 8.6% occurred at home [28]. In the current study, frequency of death at home was the highest in the Midwest, South and Northeast. Although we did not find any studies showing the causes of IE-related death at home, it is possible that this outcome is due to associated complications such as embolic phenomena and heart failure. It may also be partially explained by ill-equipped hospital facilities for admission and treatment of more complicated cases where surgery is necessary. A considerable proportion of the population in the Northeast and South live in rural areas, where access to health services is more limited due to the quality of available health resources and distance from urban centers [29]. This may also partially explain the higher frequency of deaths at home in these regions. Likewise, a considerable proportion of the population in the North lives in rural areas; however, this region had the highest proportion of IE-related deaths in younger individuals. It is therefore possible that the higher percentage of in-hospital deaths in this region was due to specific characteristics of the disease in young patients, including rheumatic heart disease, which is a predisposing condition, and infection with more virulent pathogens such as Staphylococcus aureus [29,30]. This kind of profile in younger patients requires early hospital admission to address the increased severity of these infections.

Our findings show that diseases of the circulatory system, especially heart failure, cerebrovascular diseases and hypertension, were the most frequent causes related to IE-related deaths, followed by infectious diseases, particularly sepsis. Studies investigating risk factors, trends and IE-related deaths have invariably reported causes such as heart failure and sepsis/septicemia/septic shock [22,31,32,33,34]. A study by Ahtela et al. in Finland reported that sepsis was the underlying cause of death in 11.5% of cases and other infectious diseases in 2.8% of cases. The authors also found that cardiovascular diseases were the leading non-infectious underlying cause of death and coronary artery disease and cerebrovascular disease were major contributors [28], corroborating the findings of the current study.

The ICD-10 chapter symptoms, signs and abnormal clinical and laboratory findings (not elsewhere classified) include symptoms, signs, abnormal results of clinical or other investigative procedures [17]. The categories and subcategories in this chapter were the third most frequent causes of death related to IE. The mention of these causes on death certificates is probably related to signs and symptoms detected in physical examinations and complementary tests related to the diagnosis of IE. No similar results were found in previous studies in the literature.

Mentions of diseases of the respiratory system, diseases of the genitourinary system and endocrine, nutritional and metabolic diseases may be related to complications and comorbidities that increase the risk of IE, such as renal failure and diabetes mellitus [34]. Indeed, the findings show that the most frequent causes mentioned in each of these chapters were respiratory failure, renal failure and diabetes mellitus, respectively.

The sixth most frequent chapter was external causes of morbidity and mortality, which includes abnormal reactions in patients or later complication after surgery. The most frequent category in this subcategory was prosthesis implantation. These mentions are probably related to deaths after surgery in high-risk patients. These deaths occurred predominantly in the North, which has generally poorer hospital facilities than other regions such as the Southeast and South.

Hierarchical clustering of the principal components detected by MCA resulted in three distinctive profiles of IE-related deaths.

Cluster 1 was made up predominantly of older individuals (80 years and over), women, widowers, white individuals or Asian individuals and people with little or no education, and the most frequent place of occurrence of death was at home, where the most frequent chapters were endocrine and metabolic diseases, diseases of the circulatory system and diseases of the respiratory system. The South and Southeast accounted for the highest proportion of IE-related deaths in this cluster. This profile is consistent with the sociodemographic characteristics of the South and Southeast, where the population has a high proportion of older people and is predominantly white [24]. Women form the majority of the older population since women generally live longer than men. The risk of death is higher in older people who live alone. The causes of death related to IE found in this profile are also common in this age group. As mentioned above, the most frequent subcategories in the chapters endocrine, nutritional and metabolic diseases, diseases of the circulatory system and diseases of the respiratory system were diabetes mellitus, heart failure and respiratory failure, respectively.

The second profile was made up predominantly of people aged between 30 and 79 years, men, people who were married or separated, white individuals or Asian individuals and individuals with a higher level of education. The most frequent place of occurrence of death was a hospital and the most frequent chapters were infectious diseases, genitourinary diseases, and external causes. The South and Southeast also accounted for the highest proportion of deaths in this cluster. This profile therefore complements the first profile, as deaths occurred among individuals from the same regions with different characteristics. The age of this profile is generally younger than in the first cluster, meaning that the individuals in this cluster are predominantly male, married or separated and have a higher level of education, and that the most frequent place of occurrence of death is a hospital. The most frequent causes of death in this cluster are also consistent with this profile, as deaths related to infectious diseases, renal failure [13,14,15] and prosthesis implantation are complications of IE.

The third profile differed from the others in several aspects. The cluster was made up predominantly of younger individuals (0–29 years) and accounted for all IE-related deaths in individuals aged 0–9 years. Both sexes showed similar percentages and individuals were predominantly brown/black and single. The most frequent chapter was diseases of the respiratory system, and the Northeast accounted for the highest proportion of deaths in this cluster. The profile is also consistent with the characteristics of this region, which has a higher proportion of younger and black/brown people than the Southeast and South [24]. The causes of death in ICD-10 chapters including chronic diseases are not frequent in young people. This may therefore explain the higher frequency of diseases of the respiratory system, where the most frequently mentioned condition was respiratory failure.

The main limitations of this study are the limitations inherent to SIM data. While the SIM is a comprehensive system that captures all deaths across the country, it only provides a limited set of variables, restricting the analysis to specific characteristics and mortality contexts. In addition, data quality is hampered by problems such as underreporting, inconsistencies in data entry and delays in the documentation of deaths, which can affect the accuracy and timeliness of data, particularly in remote or underserved areas [35]. Despite these limitations, SIM remains an essential resource for accessing large-scale population data, enabling robust analysis of mortality trends and patterns across Brazil.

## 5. Conclusions

The frequency of IE-related deaths over the 20-year study period was high nationally and across regions. In general, the profile of deaths was similar to that found in the literature. The method used by this study resulted in the identification of three distinctive mortality profiles, providing a better understanding of the different characteristics of IE-related deaths at the national level and across regions.

## Figures and Tables

**Table 1:** IE-related deaths in Brazil and regions according to sociodemographic characteristics, 2000–2019.

Characteristics	Brazil 52,055 ^1^	North 2168 ^1^	Northeast 9922 ^1^	Midwest 3645 ^1^	Southeast 28,114 ^1^	South 8206 ^1^	*p*-Value ^2^
Age group (years)							<0.001
0–9	1492 (2.9%)	150 (6.9%)	355 (3.6%)	95 (2.6%)	756 (2.7%)	136 (1.7%)	
10–29	4210 (8.1%)	400 (18.5%)	1509 (15.2%)	316 (8.7%)	1596 (5.7%)	389 (4.7%)	
30–59	19,003 (36.5%)	897 (41.4%)	3.887 (39.2%)	1528 (41.9%)	9939 (35.4%)	2752 (33.5%)	
60–79	20,135 (38.7%)	580 (26.8%)	2997 (30.2%)	1322 (36.3%)	11,531 (41.0%)	3705 (45.1%)	
80 and over	7215 (13.9%)	141 (6.5%)	1174 (11.8%)	384 (10.5%)	4292 (15.3%)	1224 (14.9%)	
Gender							<0.001
Female	23,593 (45.3%)	937 (43.2%)	4644 (46.8%)	1637 (44.9%)	12,896 (45.9%)	3479 (42.4%)	
Male	28,462 (54.7%)	1231 (56.8%)	5278 (53.2%)	2008 (55.1%)	15,218 (54.1%)	4727 (57.6%)	
Race/color							<0.001
Asian	336 (0.7%)	7 (0.3%)	22 (0.2%)	17 (0.5%)	263 (1.0%)	27 (0.3%)	
White	30,908 (63.6%)	604 (29.0%)	2963 (33.0%)	1.61 (50.7%)	18,491 (70.4%)	7089 (91.2%)	
Indigenous	83 (0.2%)	27 (1.3%)	9 (0.1%)	22 (0.6%)	15 (0.1%)	10 (0.1%)	
Brown	13,702 (28.2%)	1307 (62.7%)	5179 (57.7%)	1443 (41.5%)	5419 (20.6%)	354 (4.6%)	
Black	3546 (7.3%)	138 (6.6%)	804 (9.0%)	232 (6.7%)	2083 (7.9%)	289 (3.7%)	
Unknown	3480	85	945	170	1843	437	
Marital Status							<0.001
Single	12,534 (26.2%)	761 (39.9%)	3260 (38.0%)	948 (28.8%)	6162 (23.4%)	1403 (18.1%)	
Married/Consensual union	23,873 (49.9%)	876 (45.9%)	3920 (45.7%)	1613 (49.1%)	13,153 (49.9%)	4311 (55.5%)	
Legally separated	3121 (6.5%)	78 (4.1%)	296 (3.5%)	243 (7.4%)	1992 (7.6%)	512 (6.6%)	
Widowed	8338 (17.4%)	192 (10.1%)	1097 (12.8%)	483 (14.7%)	5028 (19.1%)	1538 (19.8%)	
Unknown	4189	261	1349	358	1.79	442	
Education Level							<0.001
No education	3750 (10.4%)	228 (13.7%)	1.126 (17.6%)	368 (13.6%)	1561 (8.1%)	467 (8.0%)	
1–7 years	20,353 (56.7%)	864 (51.8%)	3388 (53.0%)	1496 (55.1%)	10,888 (56.5%)	3717 (63.6%)	
8–11 years	7746 (21.6%)	418 (25.1%)	1290 (20.2%)	587 (21.6%)	4320 (22.4%)	1131 (19.4%)	
12 years or more	4037 (11.2%)	158 (9.5%)	583 (9.1%)	263 (9.7%)	2505 (13.0%)	528 (9.0%)	
Unknown	16,169	500	3535	931	8840	2363	
Place of Death							<0.001
At home	3027 (5.8%)	94 (4.3%)	735 (7.4%)	273 (7.5%)	1310 (4.7%)	615 (7.5%)	
Hospital/other health care facility	48,378 (93.0%)	2047 (94.6%)	9014 (91.0%)	3316 (91.0%)	26,554 (94.5%)	7447 (90.9%)	
Other	604 (1.2%)	23 (1.1%)	152 (1.5%)	53 (1.5%)	241 (0.9%)	135 (1.6%)	
Unknown	46	4	21	3	9	9	

Subtitles: ^1^ n (%); ^2^ Chi-squared test of independence.

## Data Availability

The data used for the investigation were taken from a public database, which can be accessed by any citizen through the DATASUS website at https://datasus.saude.gov.br/ (accessed on 25 April 2024).

## Supplementary Materials

The following supporting information can be downloaded at https://www.mdpi.com/article/10.3390/tropicalmed9120291/s1. Figure S1: Population, area and human development index of Brazilian regions, 2000 to 2019; Table S1: Cluster profile based on sociodemographic characteristics and coexisting causes of death in cases involving infectious endocarditis (IE), classified according to ICD-10 chapters. Cla/Mod represents the proportion of individuals within a specific class (cluster) relative to the total sample with that modality, while Mod/Cla denotes the proportion of individuals with a specific modality among those within a particular class (cluster).
