# Supplementary material for: Mortality Profile of Deaths Related to Infective Endocarditis in Brazil and Regions: A Population-Based Analysis of Death Records
Source: Trop Med Infect Dis. 2024 Nov 29;9(12):291. doi: 10.3390/tropicalmed9120291 (PMC11679184; doi:10.3390/tropicalmed9120291)
Supplement: Supplementary file 1 [file tropicalmed-09-00291-s001.zip › Table S1.pdf]

Table S1. Cluster profile based on sociodemographic characteristics and coexisting causes of death in cases involving infectious endocarditis (IE), classified according to ICD-10 chapters. Cla/Mod represents the proportion of individuals within a specific class (cluster) relative to the total sample with that modality, while Mod/Cla denotes the proportion of individuals with a specific modality among those within a particular class (cluster).

		Cluster 1 (N = 3,946)		Cluster 2 (N = 10,662)		Cluster 3 (N = 5,392)	
	Global (%)	Cla/Mod (%)	Mod/Cla (%)	Cla/Mod (%)	Mod/Cla (%)	Cla/Mod (%)	Mod/Cla (%)
**Age group (years)**							
0 to 9	1.2	0.0	0.0	0.0	0.0	100.0	4.5
10 to 29	8.6	0.0	0.0	1.8	0.3	98.2	31.3
30 to 59	37.2	3.1	5.8	62.4	43.6	34.5	47.6
60 to 79	38.8	21.3	41.9	68.8	50.0	9.9	14.2
80 or more	14.2	72.8	52.3	22.8	6.1	4.4	2.3
**Gender**							
Female	45.4	34.7	79.8	37.2	31.6	28.2	47.4
Male	54.6	7.3	20.2	66.7	68.4	26.0	52.6
**Race**							
Asian	0.7	31.5	1.2	63.1	0.9	5.4	0.1
White	63.5	23.7	76.1	65.3	77.8	11.0	25.9
Brown	28.5	12.0	17.3	30.1	16.1	57.9	61.1
Black	7.1	14.5	5.2	38.7	5.2	46.8	12.4
**Region**							
North	5.0	9.9	2.5	20.0	1.9	70.1	12.9
Northeast	16.4	14.6	12.1	21.3	6.5	64.1	38.9
Midwest	7.6	16.9	6.5	50.5	7.2	32.6	9.2
Southeast	54.3	21.9	60.2	61.1	62.2	17.0	34.3
South	16.9	21.9	18.7	70.4	22.3	7.7	4.8
**Marital Status**							
Single	26.9	3.9	5.2	21.6	10.9	74.6	74.3
Married/Consensual union	49.3	4.7	11.7	82.4	76.2	12.9	23.5
Legally separated	6.3	3.9	1.2	90.7	10.7	5.5	1.3
Widowed	17.6	91.9	81.8	6.7	2.2	1.4	0.9
**Education Level**							
None	10.9	37.6	20.8	16.0	3.3	46.4	18.8
1 to 7 years	57.5	20.3	59.2	54.0	58.2	25.7	54.7
8 to 11 years	20.4	13.1	13.5	57.6	22.0	29.4	22.2
12 years or more	11.2	11.3	6.5	78.3	16.5	10.4	4.3
**Place of Death**							
Hospital/Other healthcare facility	92.1	18.3	85.2	55.0	95.0	26.7	91.4
Home	6.5	41.2	13.7	31.2	3.8		
**Chapters of ICD-10**							
Endocrine, nutritional, and metabolic diseases	8.8	29.6	13.3			15.0	4.9
External causes of morbidity and mortality	15.1	12.2	9.4	66.6	18.9	21.2	11.9
Genitourinary system diseases	22.7	18.6	21.4	61.4	26.2	19.9	16.8
Infectious diseases	38.6	17.5	34.2	57.7	41.8	24.8	35.5
Circulatory system diseases	63.0	22.1	70.7	52.3	61.8	25.6	59.7
Respiratory system diseases	25.7	25.4	33.0	46.3	22.3	28.3	26.9
