# Supplementary figures and images for: Mortality Profile of Deaths Related to Infective Endocarditis in Brazil and Regions: A Population-Based Analysis of Death Records
Source: Trop Med Infect Dis. 2024 Nov 29;9(12):291. doi: 10.3390/tropicalmed9120291 (PMC11679184; doi:10.3390/tropicalmed9120291)

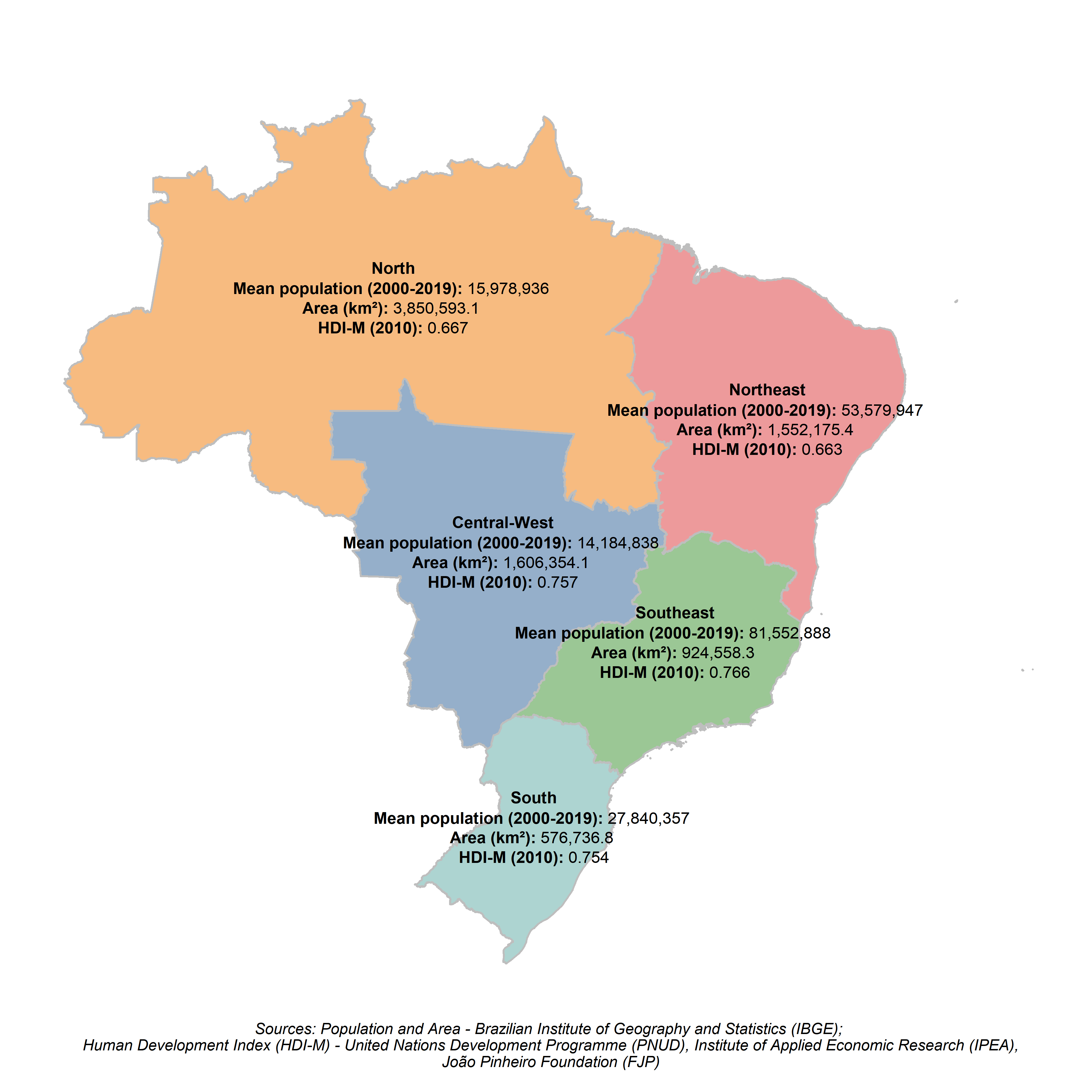

Supplement: Supplementary file 1 [file tropicalmed-09-00291-s001.zip › Figure S1.png]
